# Supplementary material for: Latent evolution of biofilm formation depends on life-history and genetic background
Source: NPJ Biofilms Microbiomes. 2023 Aug 3;9:53. doi: 10.1038/s41522-023-00422-3 (PMC10400614; doi:10.1038/s41522-023-00422-3)
Supplement: Supplementary file 1 — Supplemental Material [file 41522_2023_422_MOESM1_ESM.pdf]

SUPPLEMENTAL MATERIAL for

Latent evolution of biofilm formation depends on life-history and genetic background

Amandine Nucci[1], Eduardo P.C. Rocha[1] and Olaya Rendueles[1]*

[1] Institut Pasteur, Université Paris Cité, CNRS, UMR3525, Microbial Evolutionary Genomics, F-75015, Paris, France.
*corresponding author: olaya.rendueles-garcia@pasteur.fr

ORCID: AN, 0000-0001-7340-9075; EPCR, 0000-0001-7704-822X; OR, 0000-0002-6648-1594

Table of Contents

SUPPLEMENTARY FIGURES 2
SUPPLEMENTARY TABLES 9
REFERENCES 13

SUPPLEMENTARY FIGURES

A

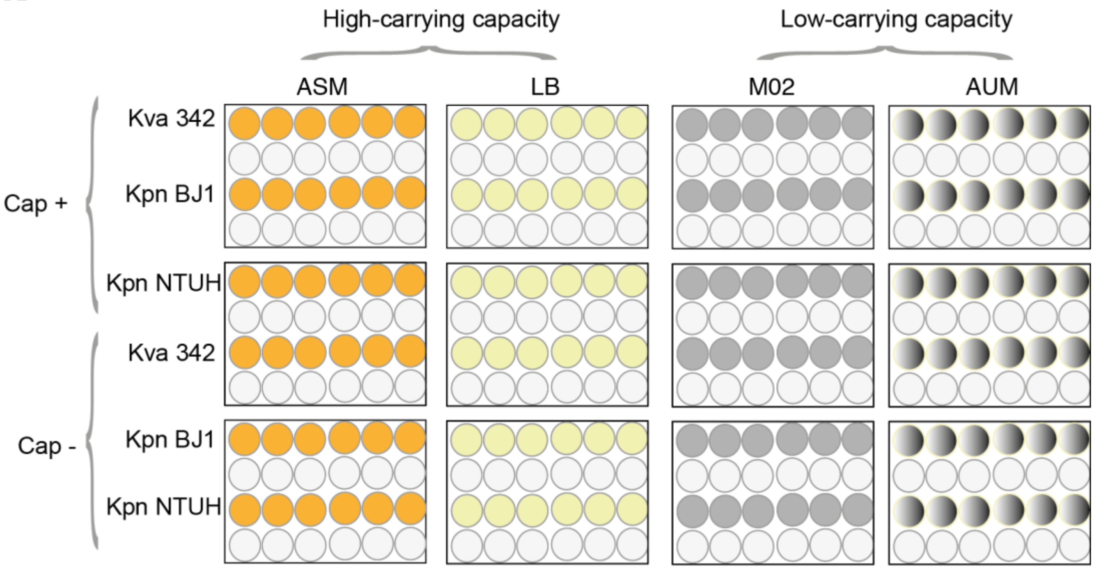

			# Populations analyzed
Environment	High carrying capacity (N = 72)	ASM	36
		LB	36
	Low carrying capacity (N = 69)	M02	33
		AUM	36
Ancestral Genotype	Capsule genotype	Cap +	69
		Cap -	72
	Strain	Kva 342	47
		Kpn NTUH	48

Supplementary Figure 1. Schematic representation of the evolution experiment. **A.** Six replicate populations of each genotype were serially passaged over 102 days. After 24 hours at 37° in static conditions, vigorous homogenisation by pipetting was applied to each population and 1% (20µL) was transferred into a new well containing 1980 µL of fresh media. One empty row was left between strains to avoid cross-contaminations. For the purpose of this study, the soil environment included in the original evolution experiment [1] was not examined as biofilm formation in soil is below the limit of detection of the assay. **B**. The table indicates the total number of populations in each group analyzed in this study. Three non-capsulated populations in AUM were excluded from the study due to contaminations of the samples [1].

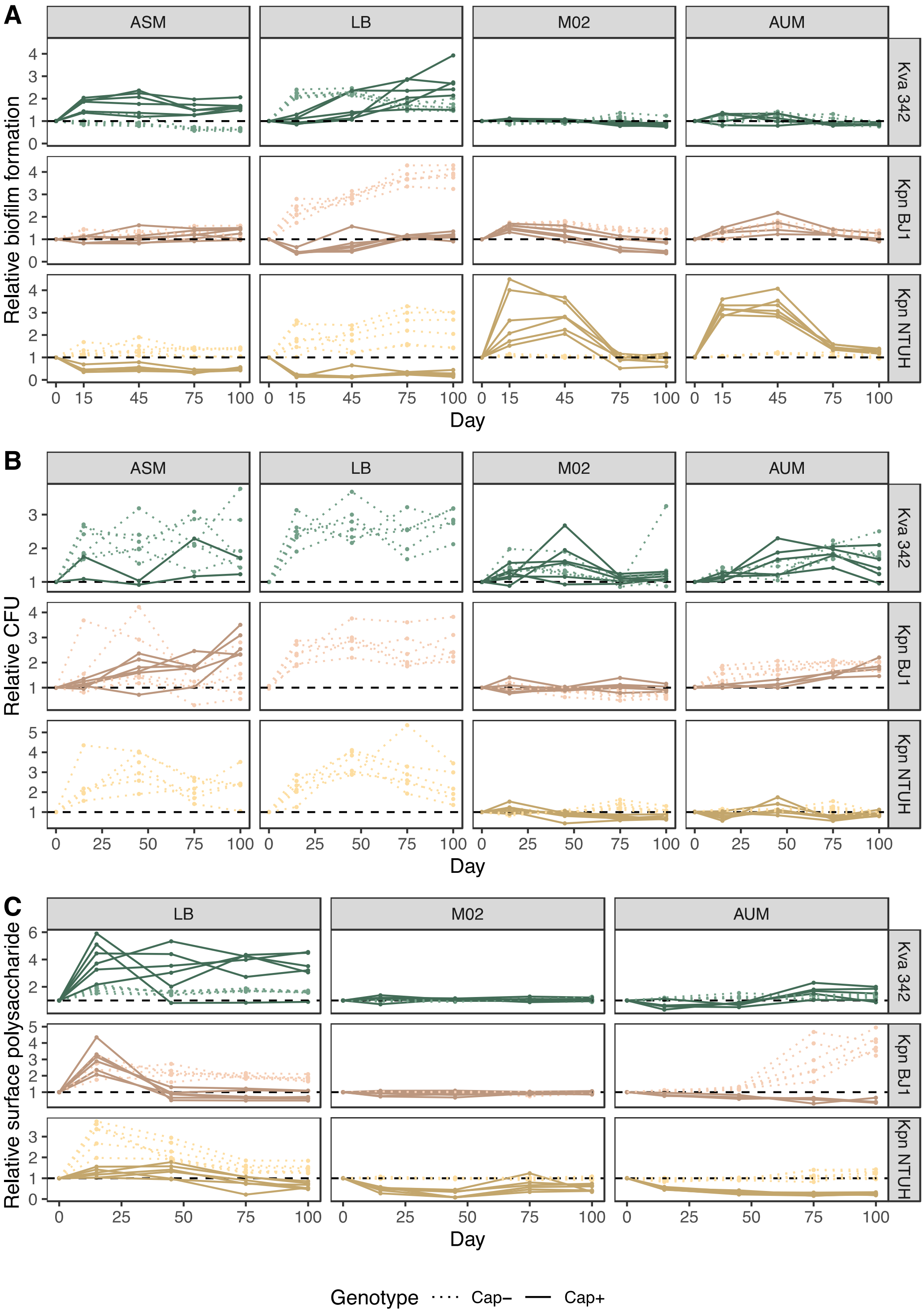

Supplementary Figure 2. Changes in biofilm formation (A), population yield (B) and production of surface polysaccharides (C), relative to the ancestor. Biofilm formation, population yield and surface polysaccharide production of each individual evolving population was tested, relative to its ancestor. Measures were taken at day 15,45, 75 and 102. Each point is the average of at least three independent biological replicates. Error bars are not shown for visualization purposes. Population yield could not be assessed in some capsulated populations

in high-carrying capacity environments (ASM & LB) because populations were hypermucoviscous, precluding any reliable assessment [1]. Surface polysaccharides in ASM could not be measured as some components of ASM interfered with the measurements. However, the presence of such components varied across time and populations (probably due to bacterial consumption) and thus surface polysaccharides could not be compared across populations.

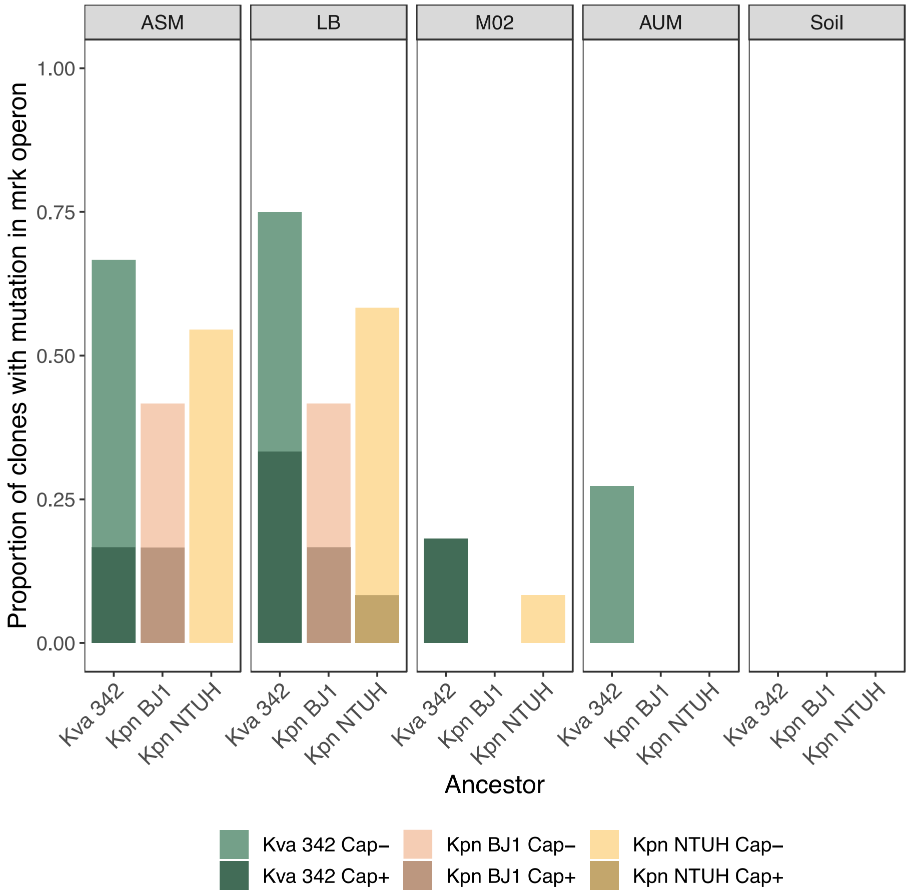

Supplementary Figure 3. Proportion of individual clones with mutations in the *mrk* operon across ancestors and environments.

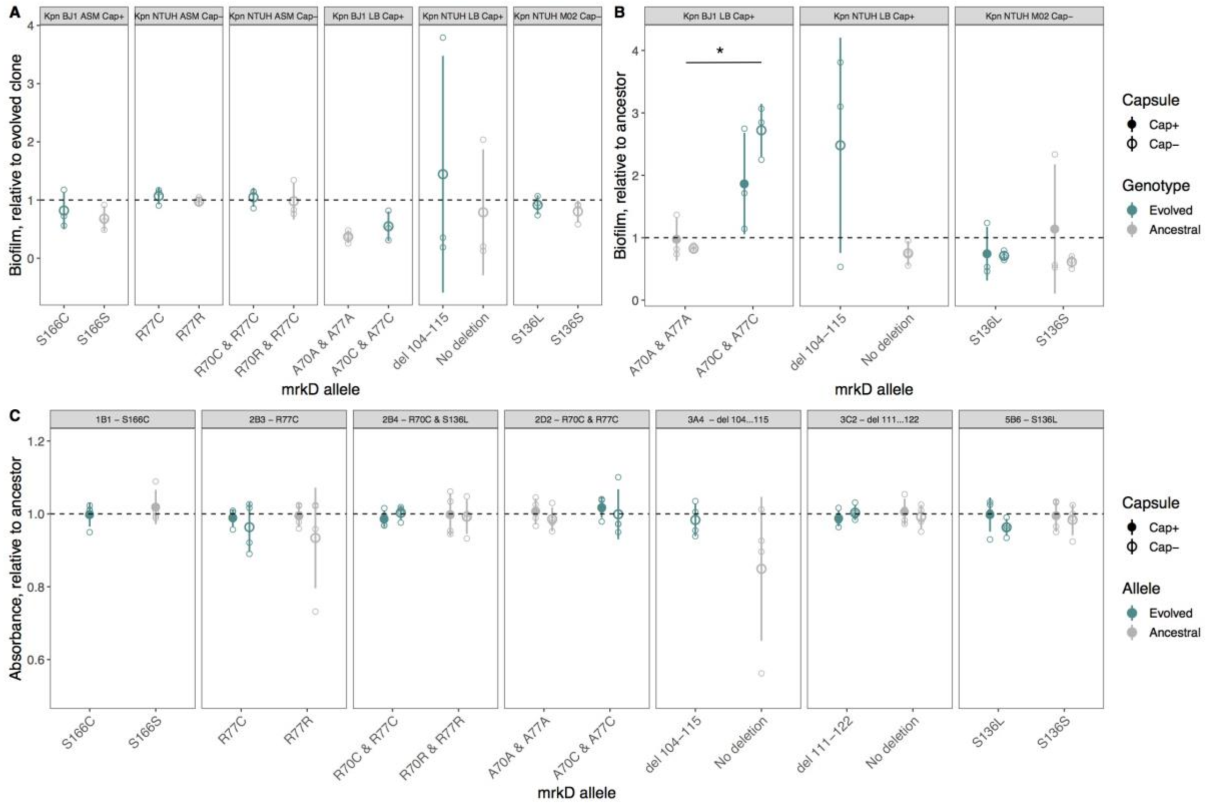

Supplementary Figure 4. Effect of *mrkD* mutations in *K. pneumoniae* BJ1 and NTUH on biofilm formation (A and B) and aggregation (C). Biofilm formation was assessed in the evolutionary treatment in which these mutations emerged (ASM, LB or M02). Full large points represent the average of capsulated clones and empty points represent non-capsulated clones. Grey points represent ancestral allele whereas green points indicate evolved alleles. Statistical analysis was performed to compare evolved vs ancestral alleles, two-sided paired t-test. Only significant comparisons are indicated * P<0.05.

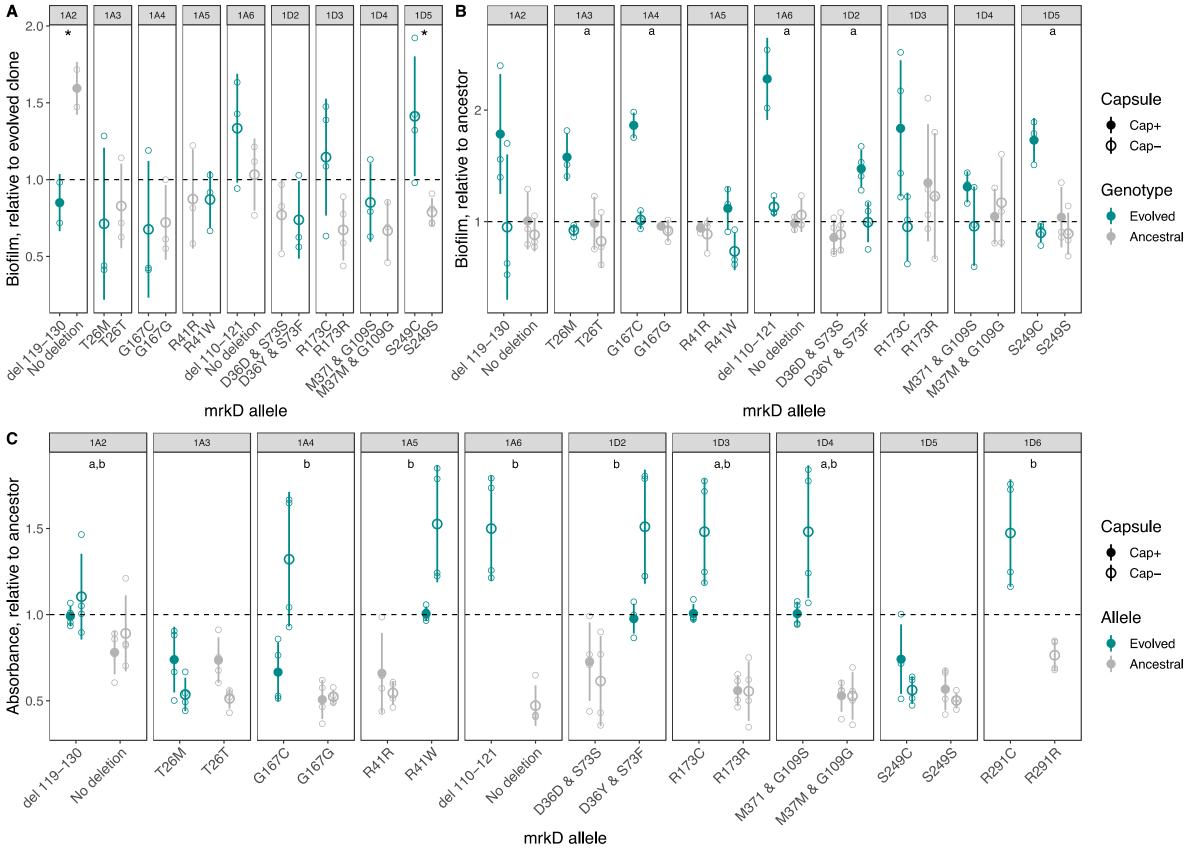

Supplementary Figure 5. Effect of *mrkD* mutations in *K. variicola* 342 on biofilm formation (A and B), aggregation (C). Panels A represents reversion of evolved allele into ancestral allele in the evolved clones. Of note, some reverted clones from originally capsulated populations are non-capsulated (1A3,1A4,1A5 and 1A6). Panels B and C represent insertion of evolved allele in ancestral backgrounds (capsulated and non-capsulated backgrounds). Full large points represent the average of capsulated clones and empty point represent non-capsulated points. Grey points represent ancestral allele whereas green indicate evolved alleles. Small individual points indicate independent experiments. Statistical analysis was performed to compare evolved vs ancestral alleles, two-sided paired T-test. 'a' indicates P<0.05 across capsulated clones whereas 'b' indicates P<0.05 across non-capsulated clones. When only one comparison was necessary * P<0.05, **P<0.01. Only significant comparisons are indicated.

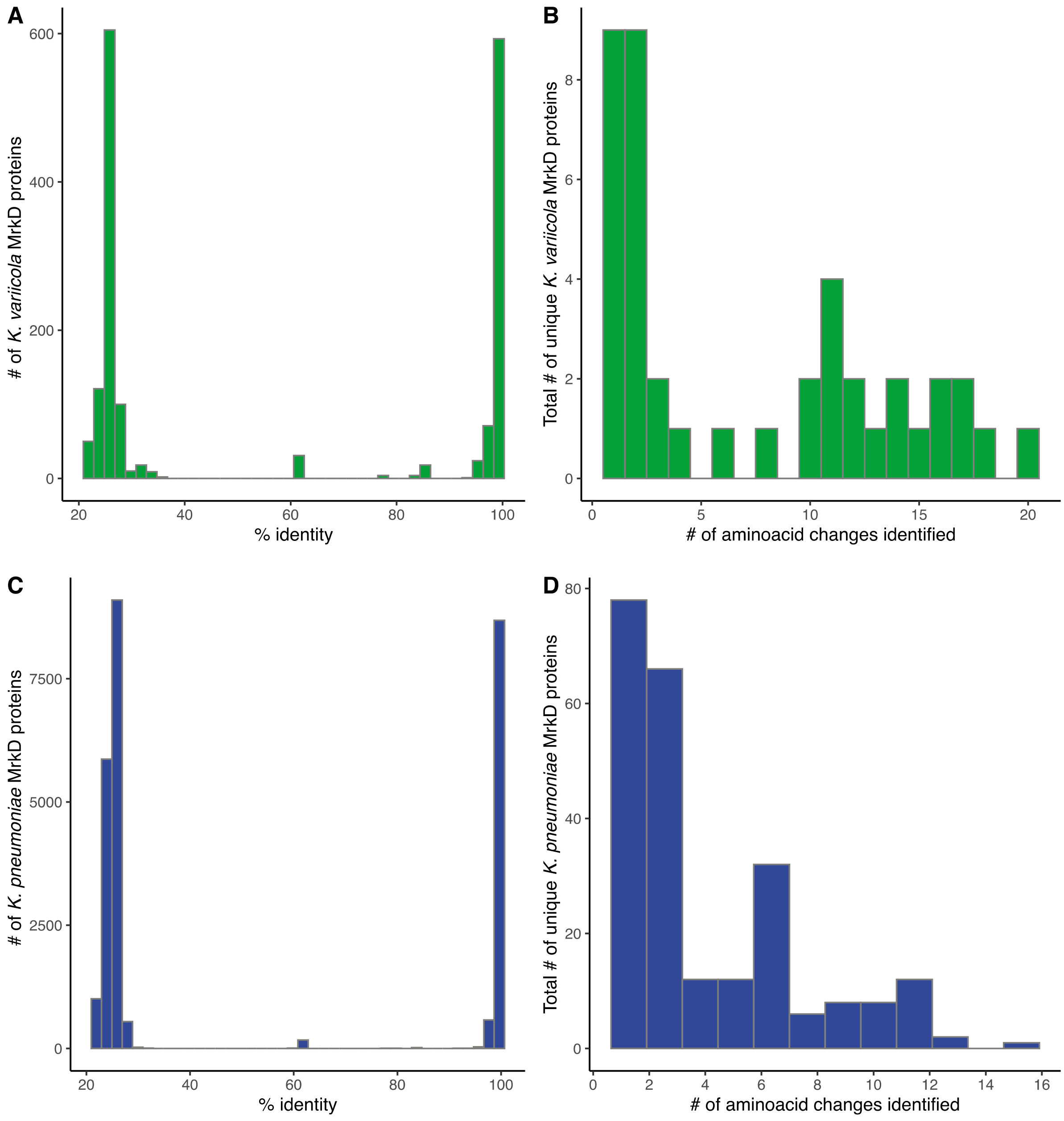

Supplementary Figure 6. Diversity of *mrkD* alleles in genomic databases. Distribution of the protein sequence identity between all proteins found in the databases for *K. variicola* (**A**) and *K. pneumoniae* (**C**). Proteins were identified by pblast with an e-value of less that 10^{-5}. Only proteins with more than 90% identity were selected for further study. The total number of aminoacid differences between our ancestral sequences and those found in the databases are depicted for *K. variicola* (**B**) and the *K. pneumoniae* (**D**).

SUPPLEMENTARY TABLES

Supplementary Table 1. Changes in population yield and surface polysaccharide production relative to the ancestor. Statistics correspond to One-sample Wilcoxon Rank Sum test. CI95 indicates the interval of confidence. Highlighted in italics are those traits that did not significantly increase, relative to the ancestor.

Day	Trait	Carrying capacity	N	Median	Mean	CI95	P-value
15	Relative CFU	Low	69	1.13	1.15	0.06	<0.001
15	Relative CFU	High	44	1.96	2	1.06	<0.001
15	Relative surf. polysacch	Low	69	0.91	0.88	0.06	0.002
15	Relative surf. polysacch	High	36	2.55	2.63	0.39	<0.001
45	Relative CFU	Low	69	1.17	1.23	0.13	<0.001
45	Relative CFU	High	44	2.54	2.54	0.31	<0.001
45	Relative surf. polysacch	Low	69	0.9	0.85	0.07	0.013
45	Relative surf. polysacch	High	36	1.77	1.9	0.3	<0.001
75	Relative CFU	Low	69	1.22	1.24	0.13	0.001
75	Relative CFU	High	44	2.11	2.14	0.22	<0.001
75	*Relative surf. polysacch*	*Low*	69	*1*	*1.1*	*0.09*	*0.981*
75	Relative surf. polysacch	High	36	1.53	1.7	0.33	<0.001
100	Relative CFU	Low	69	1.17	1.22	0.17	0.024
100	Relative CFU	High	43	2.33	2.32	0.26	<0.001
100	*Relative surf. polysacch*	*Low*	*69*	*1*	*1.2*	*0.12*	*0.929*
100	Relative surf. polysacch	High	36	1.45	1.6	0.3	<0.001

Supplementary Table 2. Mutations in *mrk* operon in clones sequenced at the end of the evolution experiment.

Environment	Strain	Ancestor	Clone ID	Gene	Mutation	Annotation
ASM	Kva 342	Cap +	1A3	*mrkD*	C→T	T26M (ACG→ATG)
ASM	Kva 342	Cap +	1A3	*mrkD*	C→T	R291C (CGC→TGC)
ASM	Kva 342	Cap +	1A5	*mrkB*	T→C	F8S (TTC→TCC)
ASM	Kva 342	Cap +	1A5	*mrkD*	A→T	R41W (AGG→TGG)
ASM	Kva 342	Cap -	1D1	*mrkF*	IS insertion	coding (345/636 nt)
ASM	Kva 342	Cap -	1D1	*mrkC*	G→A	G439E (GGG→GAG)
ASM	Kva 342	Cap -	1D2	*mrkD*	G→T	D36Y (GAC→TAC)
ASM	Kva 342	Cap -	1D2	*mrkD*	C→T	S73F (TCC→TTC)
ASM	Kva 342	Cap -	1D3	*HJIKBMFB_00805 / mrkF*	G→T	intergenic (-600/-106)
ASM	Kva 342	Cap -	1D3	*mrkD*	C→T	R137C (CGC→TGC)
ASM	Kva 342	Cap -	1D4	*mrkF*	G→T	Q156H (CAG→CAT)
ASM	Kva 342	Cap -	1D4	*mrkD*	G→T	M37I (ATG→ATT)
ASM	Kva 342	Cap -	1D4	*mrkD*	G→A	G109S (GGC→AGC)
ASM	Kva 342	Cap -	1D5	*mrkD*	A→T	S249C (AGC→TGC)
ASM	Kva 342	Cap -	1D6	*mrkD*	C→T	R291C (CGC→TGC)
ASM	Kpn BJ1	Cap +	1B1	*mrkD*	T→A	S166C (AGC→TGC)
ASM	Kpn BJ1	Cap +	1B5	*mrkD*	T→A	S151C (AGC→TGC)
ASM	Kpn BJ1	Cap -	2A1	*mrkD*	Δ12 bp	coding (111-122/996 nt)
ASM	Kpn BJ1	Cap -	2A4	*mrkD*	Δ12 bp	coding (111-122/996 nt)
ASM	Kpn BJ1	Cap -	2A5	*mrkD*	T→A	S166C (AGC→TGC)
ASM	Kpn NTUH	Cap -	2B1	*mrkD*	New junction	+70bp
ASM	Kpn NTUH	Cap -	2B2	*mrkD*	C→A	G263C (GGC→TGC)
ASM	Kpn NTUH	Cap -	2B3	*mrkD*	G→A	R77C (CGC→TGC)
ASM	Kpn NTUH	Cap -	2B4	*mrkD*	G→A	S136L (TCG→TTG)
ASM	Kpn NTUH	Cap -	2B4	*mrkD*	G→A	R70C (CGC→TGC)
ASM	Kpn NTUH	Cap -	2B5	*mrkD*	C→A	G228C (GGC→TGC)
ASM	Kpn NTUH	Cap -	2B5	*mrkD*	C→A	D58Y (GAC→TAC)
ASM	Kpn NTUH	Cap -	2B6	*mrkD*	G→A	S185L (TCG→TTG)
LB	Kva 342	Cap +	2C1	*mrkD*	Δ12 bp	coding (110-121/996 nt)
LB	Kva 342	Cap +	2C3	*mrkD*	Δ12 bp	coding (110-121/996 nt)
LB	Kva 342	Cap +	2C5	*mrkD*	Δ12 bp	coding (110-121/996 nt)
LB	Kva 342	Cap +	2C6	*mrkD*	C→G	N98K (AAC→AAG)
LB	Kva 342	Cap -	3B1	*mrkF*	Δ168 bp	coding (270-437/636 nt)
LB	Kva 342	Cap -	3B2	*mrkD*	Δ12 bp	coding (110-121/996 nt)
LB	Kva 342	Cap -	3B4	*mrkF*	(A)5→4	coding (580/636 nt)
LB	Kva 342	Cap -	3B5	*mrkD*	Δ12 bp	coding (110-121/996 nt)
LB	Kva 342	Cap -	3B6	*mrkD*	Δ12 bp	coding (100-111/996 nt)
LB	Kpn BJ1	Cap +	2D3	*mrkD*	Δ12 bp	coding (111-122/996 nt)
LB	Kpn BJ1	Cap +	2D4	*mrkD*	Δ12 bp	coding (111-122/996 nt)
LB	Kpn BJ1	Cap -	3C2	*mrkD*	C→T	G109S (GGC→AGC)
LB	Kpn BJ1	Cap -	3C2	*mrkD*	Δ12 bp	coding (111-122/996 nt)
LB	Kpn BJ1	Cap -	3C3	*mrkD*	Δ12 bp	coding (98-109/996 nt)
LB	Kpn BJ1	Cap -	3C4	*mrkD*	Δ12 bp	coding (98-109/996 nt)
LB	Kpn NTUH	Cap +	3A4	*mrkD*	Δ12 bp	coding (104-115/996 nt)
LB	Kpn NTUH	Cap -	3D1	*mrkD*	(GATCCGGGGGCA)1→2	coding (131/996 nt)
LB	Kpn NTUH	Cap -	3D2	*mrkD*	(GATCCGGGGGCA)1→2	coding (131/996 nt)
LB	Kpn NTUH	Cap -	3D3	*mrkJ*	A→C	Q219P (CAG→CCG)
LB	Kpn NTUH	Cap -	3D3	*mrkD*	A→T	Y69N (TAT→AAT)
LB	Kpn NTUH	Cap -	3D4	*mrkD*	Δ12 bp	coding (104-115/996 nt)
LB	Kpn NTUH	Cap -	3D5	*mrkD*	New junction	coding (87/996 nt)
LB	Kpn NTUH	Cap -	3D6	*mrkD*	G→A	R57C (CGC→TGC)
M02	Kva 342	Cap +	4A3	*HJIKBMFB_00805 / mrkA*	G→T	intergenic (-41/-665)
M02	Kva 342	Cap +	4A6	*mrkH*	IS insertion	coding (95/705 nt)
M02	Kpn NTUH	Cap -	5B6	*mrkD*	G→A	S136L (TCG→TTG)
AUM	Kva 342	Cap -	6B2	*mrkB*	T→A	N109K (AAT→AAA)
AUM	Kva 342	Cap -	6B3	*mrkC*	T→G	L459R (CTG→CGG)
AUM	Kva 342	Cap -	6B3	*mrkF*	T→G	V35G (GTC→GGC)
AUM	Kva 342	Cap -	6B6	*mrkC*	C→A	A549E (GCG→GAG)

Supplementary Table 3. Number of clones with mutation in *mrkD*. Six independent capsulated and non-capsulated clones from each population were isolated. *mrkD* gene was amplified and Sanger sequenced. The presence or absence of mutations in *mrkD* was noted in function of the capsule background.

Population	Mutation	Day	Capsulated clones with *mrkD* evolved allele	Non-capsulated clones with *mrkD* evolved allele	Fate of the mutation
1A2	Δ 119-130nt	7	6/6	1/6	Extinct
1A3	T26M	7	4/6	0/6	Fix
1A4	G167C	7	4/6	0/6	Extinct
1A4	T139P	30	0/6	5/6	Extinct
1A5	R41W	7	0/6	6/6	Fix
1A6	Δ 110-121nt	30	0/6	5/6	Extinct

Supplementary Table 4. Primers used in this study.

Species	Name	Direction	Use	Sequence
Primers for sequencing				
K.variicola	mrkD1_24_seq5	Forward	Verify and sequence mrkD in Kva	GATGGTAAGGAGATCGGCGTTG
K.variicola	mrkD1_24_seq3	Reverse	Verify and sequence mrkD in Kva	CTTCCCGCTCATCACCGTAC
K. pneumoniae	26_56.mrkD_F_check	Forward	Verify and sequence *mrkD* in Kpn	GCAGTGTTCGGTGGATCTGG
K. pneumoniae	26_56.mrkD_R_check	Reverse	Verify and sequence *mrkD* in Kpn	TTTCGGCAATCCCTTCATTCC
	pKNG101.verif5	Forward	Verify pKNG101 plasmid & insert	CTACATATCACAACGTGCGTGG
	pKNG101.verif3_SpeIout	Reverse	Verify pKNG101 plasmid & insert	ACCAAGCCTATGCCTACAGC
Construction of mutants				
K.variicola	24.mrkD_F_gib	Forward	amplify *mrkD* in strain Kva 342 with tails for pknG101 (Gibson assembly)	cataagtagaagcagcaacccaagtagctttaccagcatcCGGTGCAGAACAACCTCACG
K.variicola	24.mrkD_R_gib	Reverse	amplify *mrkD* in strain Kva 342 with tails for pknG101 (Gibson assembly)	cttccgctcaggtccttgtcctttaacgaggattgttacCGGTCATAATTGGTCTCGAAAC
K. pneumoniae	26_56.mrkD_F_gib	Forward	amplify *mrkD* with tails for pknG101 (Gibson assembly)	cataagtagaagcagcaacccaagtagctttaccagcatcCGTGGTTAAGGTCGCGTTCG
K. pneumoniae	26_56.mrkD_R_gib	Reverse	amplify *mrkD* with tails for pknG101 (Gibson assembly)	cttccgctcaggtccttgtcctttaacgaggattgttacACGGCTGCGGTTTCATAAACG
	pkng101.F gibson	Forward	Linearise & amplify suicide vector pKNG101	GATGCTGGTAAAGCTACTTG
	pkng101.R gibson	Reverse	Linearise & amplify suicide vector pKNG101	GTAACAATCCTCGTTAAAGGAC

REFERENCES

1. Nucci, A., E.P.C. Rocha, and O. Rendueles, *Adaptation to novel spatially-structured environments is driven by the capsule and alters virulence-associated traits.* Nature Communications, 2022. **13**(1): p. 4751.
